# Development and Validation of a Risk Stratification Model Using Disease Severity Hierarchy for Mortality or Major Cardiovascular Event

**DOI:** 10.1001/jamanetworkopen.2020.8270

**Published:** 2020-07-17

**Authors:** Che Ngufor, Pedro J. Caraballo, Thomas J. O’Byrne, David Chen, Nilay D. Shah, Lisiane Pruinelli, Michael Steinbach, Gyorgy Simon

**Affiliations:** 1 Division of Digital Health Science, Department of Health Science Research, Mayo Clinic, Rochester, Minnesota; 2 The Robert D. and Patricia E. Kern Center for the Science of Health Care Delivery, Mayo Clinic, Rochester, Minnesota; 3 Division of General Internal Medicine, Department of Internal Medicine, Mayo Clinic, Rochester, Minnesota; 4 Division of Healthcare Policy and Research, Department of Health Science Research, Mayo Clinic, Rochester, Minnesota; 5 University of Minnesota School of Nursing, Minneapolis; 6 Department of Computer Science and Engineering, University of Minnesota, Minneapolis; 7 Institute for Health Informatics, University of Minnesota, Minneapolis; 8 Department of Medicine, University of Minnesota, Minneapolis

## Abstract

**Importance:**

Clinical domain knowledge about diseases and their comorbidities, severity, treatment pathways, and outcomes can facilitate diagnosis, enhance preventive strategies, and help create smart evidence-based practice guidelines.

**Objective:**

To introduce a new representation of patient data called disease severity hierarchy that leverages domain knowledge in a nested fashion to create subpopulations that share increasing amounts of clinical details suitable for risk prediction.

**Design, Setting, and Participants:**

This retrospective cohort study included 51 969 patients aged 45 to 85 years, with 10 674 patients who received primary care at the Mayo Clinic between January 2004 and December 2015 in the training cohort and 41 295 patients who received primary care at Fairview Health Services from January 2010 to December 2017 in the validation cohort. Data were analyzed from May 2018 to December 2019.

**Main Outcomes and Measures:**

Several binary classification measures, including the area under the receiver operating characteristic curve (AUC), Gini score, sensitivity, and positive predictive value, were used to evaluate models predicting all-cause mortality and major cardiovascular events at ages 60, 65, 75, and 80 years.

**Results:**

The mean (SD) age and proportions of women and white individuals were 59.4 (10.8) years, 6324 (59.3%) and 9804 (91.9%), respectively, in the training cohort and 57.4 (7.9) years, 21 975 (53.1%), and 37 653 (91.2%), respectively, in the validation cohort. During follow-up, 945 patients (8.9%) in the training cohort died, while 787 (7.4%) had major cardiovascular events. Models using the new representation achieved AUCs for predicting death in the training cohort at ages 60, 65, 75, and 80 years of 0.96 (95% CI, 0.94-0.97), 0.96 (95% CI, 0.95-0.98), 0.97 (95% CI, 0.96-0.98), and 0.98 (95% CI, 0.98-0.99), respectively, while standard methods achieved modest AUCs of 0.67 (95% CI, 0.55-0.80), 0.66 (95% CI, 0.56-0.79), 0.64 (95% CI, 0.57-0.71), and 0.63 (95% CI, 0.54-0.70), respectively.

**Conclusions and Relevance:**

In this study, the proposed patient data representation accurately predicted the age at which a patient was at risk of dying or developing major cardiovascular events substantially better than standard methods. The representation uses known relationships contained in electronic health records to capture disease severity in a natural and clinically meaningful way. Furthermore, it is expressive and interpretable. This novel patient representation can help to support critical decision-making, develop smart guidelines, and enhance health care and disease management by helping to identify patients with high risk.

## Key Points

**Question:**

Does incorporating clinical domain knowledge regarding diseases, disease severity, and treatment pathways into machine learning improve risk stratification?

**Findings:**

In this retrospective cohort study involving 51 969 patients, a new representation of patient data was developed and used to train machine learning models to predict mortality and major cardiovascular events. Results showed substantial improvement in prediction performance compared with traditional patient data representation methods.

**Meaning:**

The findings of this study suggest that methods that can extract and represent the clinical knowledge contained in electronic medical records should be incorporated into machine learning models for use in clinical decision support systems.

## Teaser

This cohort study introduces a new representation of patient data called disease severity hierarchy that leverages domain knowledge in a nested fashion to create subpopulations that share increasing amounts of clinical details suitable for risk prediction.

## Introduction

More than one-quarter of US adults have multiple (ie, ≥2) chronic conditions (MCCs), with a prevalence that increases with age.^1,2,3^ Patients with MCCs use more health care resources, incur high costs of care, and face greater mortality, with worse quality of life.^3,4,5^ However, identifying, treating, and improving health outcomes of patients with MCCs have been hindered by the marked complexity and heterogeneity of this population. Current comparative effectiveness research and evidence-based practice guidelines (EBPGs) often focus on 1 disease at a time or on highly prevalent co-occurring conditions, with risk stratification based on simple comorbidity counts.^6^ However, patients with MCCs represent complex cases and could benefit from smarter EBPGs that leverage data-driven machine learning and data about disease interactions, disease severity, and treatment pathways.

Predictive analytics based on electronic health records (EHRs) can provide crucial information for making better clinical decisions about patients with MCCs. However, it also raises important questions about the appropriate representation of MCCs for predicting health outcomes. Current methods tend to focus on traditional comorbidity representations, such as simple summaries of individual conditions (eg, counts, sums, and most recent values) or aggregate measures of comorbidities.^6^ Unfortunately, these representations do not reflect an individual’s comorbid disease history and severity and do not account for interactions with different treatment pathways. Therefore, this leaves a large gap in our knowledge regarding how to optimally manage complex MCCs.^7^

To address this critical knowledge gap, we introduce a novel representation of patient data, disease severity hierarchy (DSH), which explores diseases and their known treatment pathways in a nested fashion. As the DSH tree is traversed from the root to the leaves, subpopulations of patients with similar clinical characteristics, such as disease severity, progression, and survival, are created, thereby providing discriminative features suitable for developing risk stratification models. To quantitatively represent the information embedded in DSH, we further introduce a risk scoring system, which encodes disease severity and its intensification as numeric values along any path of the DSH tree. We then test the effectiveness of DSH in the case of type 2 diabetes and its comorbidities (ie, hypertension, obesity, and hyperlipidemia) in predicting all-cause mortality (ACM) and major cardiovascular events (MCEs).

## Methods

This study was reviewed and approved by the Mayo Clinic institutional review board as a minimal risk study, and informed consent was not required. The study conforms to the Strengthening the Reporting of Observational Studies in Epidemiology (STROBE) reporting guideline.^8^

### Study Source and Population

**Figure 1.**
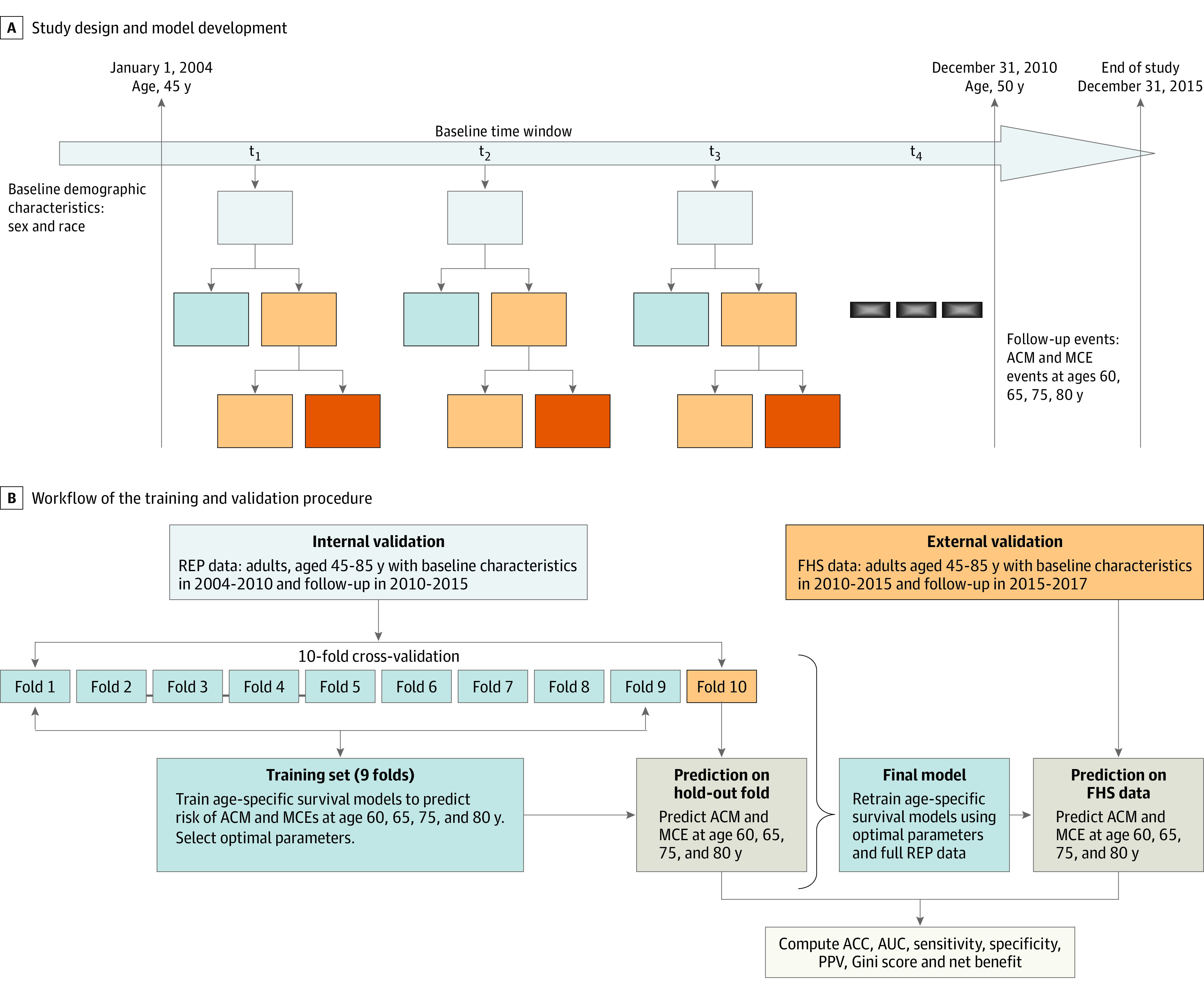
Study Design and Model Development A, Overview of study design with disease severity hierarchy determined at each time point in the baseline time window. B, Workflow of the training and validation procedure and selection of final model. Data were randomly divided in 10 equal and independent parts (ie, folds), then the models were trained on 9 folds and tested on the hold-out fold. The procedure was repeated until each fold was used for testing. At each step, optimal parameters were selected and performance was evaluating on the hold-out fold. The final model was then developed on the complete Rochester Epidemiology Project (REP) data using optimal parameters. ACC indicates accuracy; ACM, all-cause mortality; AUC, area under the receiver operating characteristic curve; FHS, Fairview Health Services; MCE, major cardiovascular event; and PPV, positive predictive value.

We retrospectively analyzed the medical records of 10 674 adults, aged 45 to 85 years, included in the Rochester Epidemiology Project (REP) database who received primary care at the Mayo Clinic from January 1, 2004, to December 31, 2015, to develop the models.^9^ The models were externally validated using data for 41 295 patients, aged 45 to 85 years, who had a primary care visit between January 1, 2010, and December 31, 2017, at Fairview Health Services (FHS).^10^ Patients in the REP data entered the study at their age on January 1, 2004 (index date), and were still alive on December 31, 2010. These patients were followed up until December 31, 2015. Correspondingly, patients in the FHS data were included in the analysis if they were aged at least 45 years on January 1, 2010 (index date), and were alive on December 31, 2015. The FHS patients were followed up until December 31, 2017. We defined the period between the index date and start of follow-up as the baseline time window (BTW). Figure 1A shows an overview of the study design.

### Outcomes

The primary outcome was defined as the patient’s age-time of ACM and MCE in the follow-up period. Patients entered the study at age 45 years, were left truncated at age 51 years (REP) and age 50 years (FHS) when they were first classified as at risk, and exited the study at the event (ACM or MCE) or censored. The left truncation allowed us to put patients in the risk set only when they were actually under follow-up by the study.^11,12,13^ The use of the age-time scale provided an expressive and flexible way to control the effect of age, especially for older adults. It also provided a relatively meaningful basis on which to examine how risk varies over time.^11,12,13^

Death records in the REP and FHS were obtained from the Minnesota Electronic Death Certificates and the National Death Index. We defined MCE as a composite of myocardial infarction, stroke, percutaneous transluminal coronary angioplasty, use of cardiac devices, coronary artery procedures, congestive heart failure, ischemic heart disease, coronary artery disease, cardiomyopathy, cardiac arrest, or angina occurring in the follow-up period. The list of *International Classification of Diseases, Ninth Revision *(*ICD-9*) and *International Statistical Classification of Diseases, Tenth Revision, Clinical Modification *(*ICD-10-CM*) codes for these conditions can be found in the eAppendix in the Supplement. The REP and FHS have robust data quality standards, so missing values were low, and deaths and MCE records were complete in this study.

### Covariates

Baseline demographic variables used for the analysis included sex and race/ethnicity. Comorbidities of type 2 diabetes, laboratory results, vital signs, and medication use were ascertained from the index date to the start of follow-up.

### Type 2 Diabetes and Its Comorbidities

A patient was considered to have type 2 diabetes and its comorbidities if at least 1 of the following indications was present during BTW: (1) type 2 diabetes, clinical diagnosis (based on *ICD-9* and *ICD-10-CM* codes), fasting blood glucose of at least 126 mg/dL (to convert to millimoles per liter, multiply by 0.0555), random blood glucose level of at least 200 mg/dL, or glycated hemoglobin (HbA_1c_) level of at least 6.5% (to convert to proportion of total hemoglobin, multiply by 0.01)^14^; (2) hypertension, clinical diagnosis or blood pressure at least 140/90 mm Hg; (3) hyperlipidemia, low-density lipoprotein cholesterol of at least 100 mg/dL (to convert to millimoles per liter, multiply by 0.0259); and (4) obesity, body mass index (calculated as weight in kilograms divided by height in meters squared) of at least 30.

### Summarizing Time-Varying Covariates

The comorbidities, laboratory results, vital signs, and medications were captured throughout BTW; as such, a patient may have repeated measurements of the same variable. These potentially correlated measurements present a major challenge for conventional machine learning methods.^15,16^ Therefore, we aggregated the repeated observations for each patient to create cross-sectional data. We created 2 comorbidity variables (the rolling comorbidity,^17^ ie, the condition persisted throughout the BTW, and the most frequent value), 2 medication variables (the number of times [frequency] the patient received any medication class and an indicator for the presence or absence of any class), and 3 variables for laboratory results and vital signs (frequency, median, and last observed value in BTW).

No observable association was found in the patterns of missing values (laboratory results and vital signs) in the data (eFigure 1 and eFigure 2 in the Supplement). Missing laboratory results and vital signs (with <35% missingness) were imputed using the random forest imputation method.^18^

### DSH

Diabetes and other heterogeneous diseases have multiple underlying molecular mechanisms that are reflected in varying sets of comorbidities.^19^ The heterogeneity can be largely viewed as manifestations of different disease mechanisms, which are present in different subpopulations and in different proportions. Unfortunately, the underlying disease mechanisms are not known a priori. The proposed DSH is designed to address how heterogeneous diseases, such as diabetes, can be modeled without explicitly knowing the underlying disease mechanisms. It accomplishes this goal by transforming the multifaceted heterogeneous EHR data into a clinically meaningful patient state representation that uniquely captures and encodes known relationships about disease severity, treatment pathways, and outcomes. For each clinical encounter, we extracted diagnoses, laboratory results, vital signs, and interventions to construct the DSH representing the patient’s health trajectory as a sequence of clinical findings.

#### Clinical Findings and Actions

We considered a patient’s health trajectory a sequence of clinical findings and actions triggered by these findings. For instance, a new laboratory result (eg, a fasting blood glucose level of 130 mg/dL) and the corresponding clinical context describing the disease, its severity, symptoms, medications, interventions, or outcomes observed during a clinical visit represent a clinical finding. After examining the finding, an action may be triggered (eg, introducing a new diabetic medication).

**Figure 2.**
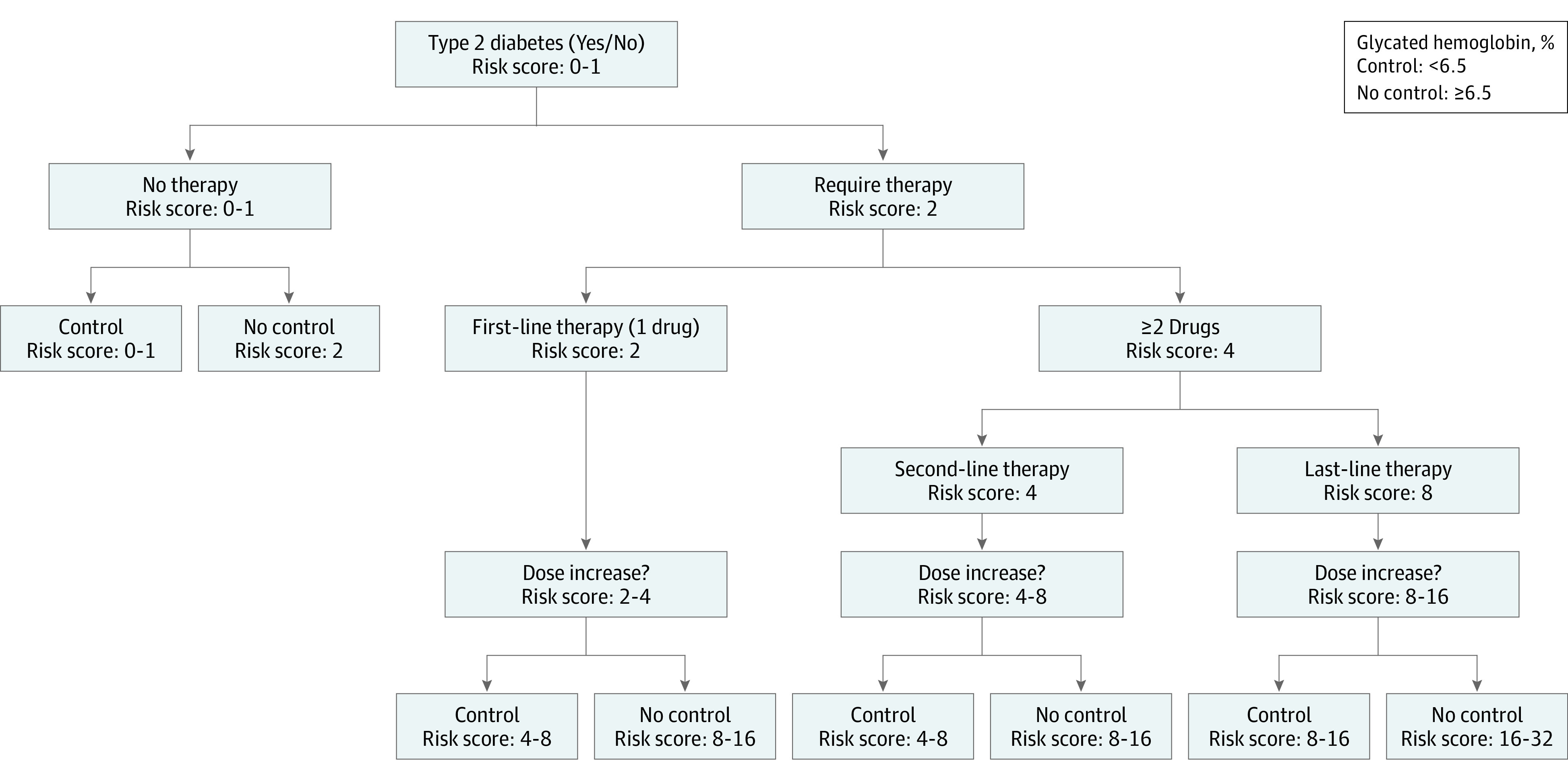
Disease Severity Hierarchy for Type 2 Diabetes An example of a 6-level disease severity hierarchy tree structure and corresponding risk score allocations for type 2 diabetes. Disease severity intensifies on any right branch from the root to the leaves. A patient is considered under control if the laboratory result or vital sign associated with the condition is within its predefined reference range, eg, glycated hemoglobin less than 6.5% (to convert to proportion of total hemoglobin, multiply by 0.01). The disease severity hierarchy structure for hypertension and hyperlipidemia are similar to that for type 2 diabetes. An example of the disease severity hierarchy tree for obesity is presented in eFigure 3 in the Supplement.

The sequence of clinical findings can be described at different granular levels: disease presence or absence, untreated disease, disease requiring therapy, aggressiveness of therapy (first-line, second-line, or last-line therapy), dose increase or decrease, and disease control status. Thus, DSH captures the patient’s health status, disease severity, treatment pathway, and outcomes, which can be represented as a binary tree. Figure 2 depicts a DSH representation for type 2 diabetes, and eFigure 3 in the Supplement depicts a DSH representation for obesity.

#### Modeling Relationships in EHR

Data from EHRs are rich with information on disease conditions, laboratory results, vital signs, medications, and treatment pathways. Unfortunately, most existing methods treat this information as independent and do not take advantage of preexisting domain knowledge about relationships. In contrast, DSH concisely captures this expert knowledge. Consider a patient taking metformin and sulfonylurea with an HbA_1c_ of 6%. At the sixth level of the DSH tree (Figure 2), the patient is controlled; at the fourth, is receiving second-line therapy; at the first, has diabetes.

### DSH Risk Score

In traversing the DSH tree from the root to the leaves, we capture disease severity beneath each node; the right branch leads to intensification of the disease (eg, ≥2 drugs) and the left branch leads to a steady or controlled state (eg, first-line therapy) . Motivated by this natural flow of information, we proposed a simple risk scoring system to quantitatively represent the information embedded in DSH. Specifically, we assigned risk scores to the nodes so that the score on the right branch doubles that of the node above, while the score on the left brach is the same as that of the node above. The risk score of the root node is either 1 (with disease) or 0 (disease free). We aggregated the time-varying DSH risk scores in the BTW into 3 DSH risk variables, as follows: sum, mean, and last observed value.

### Statistical Analysis

We report descriptive statistics of patient characteristics, with means and SDs for continuous variables and counts and percentages for categorical variables, and of the performance of the models. We developed age-specific survival models, in which we used the patient’s age instead of the traditional time-on-study as the scale for predicting the risk of ACM and MCE at ages 60, 65, 75, and 80 years based on DSH risk scores, while adjusting for sex and race/ethnicity. We trained the regularized Cox proportional hazard (CPH) regression model through 10-fold cross-validation.^20^ All data analyses were performed using R version 3.5.1 (R Project for Statistical Computing). No prespecified level of statistical significance was set.

#### Training and Validation

A regularized CPH algorithm requires the selection of 2 tuning parameters to avoid overfitting, as follows: (1) the least absolute shrinkage and selection operator (lasso) penalty and (2) the ridge penalty.^21,22^ We set up a grid for each combination and selected the best in 10-fold cross-validation. The final model was developed on the entire REP using the best parameters and validated externally on FHS (Figure 1B).

#### Performance Measures

We computed the accuracy, receiver operating characteristic curve, area under the receiver operating characteristic curve (AUC), cumulative gain curve and Gini score,^23^ sensitivity, specificity, and positive predictive value (PPV) at each age-time point using methods appropriate for censored data.^24^ The clinical utility of the models was evaluated using the net benefit computed across classification thresholds.^25,26^ The mean and 95% CIs of the performance scores over the cross-validations on REP data and point estimates on FHS data are reported. We show the association between sensitivity, PPV, and prediction density graphically with respect to the threshold.

#### Model Comparison

Because DSH captures laboratory results and medications associated with type 2 diabetes and its comorbidities, we conducted experiments to compare its predictive ability with that of the individual comorbidities, medications, laboratory results, and vital signs. Specifically, we compared the performance of models trained using the following group of predictors: DSH risk scores only (DSH-RS), comorbidities with medications (COM), and COM with laboratory results and vital signs (COM + LB/VS). We aimed to show that DSH-RS outperforms COM and had similar or better performance compared with COM + LB/VS.

## Results

### Baseline Characteristics

We implemented a 4-level DSH tree, in which the maximum allocated DSH risk score was 8. Table 1 presents descriptive statistics for the REP (10 674 participants) and FHS (41 295 participants) data. The REP data contained older patients and more women than the FHS data (mean [SD] age, 59.4 [10.8] years vs 57.4 [7.9] years; 6324 [59.3%] women vs 21 975 [53.2%] women). Both data sets comprised primarily white patients (REP, 9804 [91.9%]; FHS, 37 653 [91.2%]). During follow-up, 945 patients (8.9%) in the REP cohort died, while 787 (7.4%) had an MCE. Correspondingly, in the FHS cohort, 1857 (4.5%) died and 1857 (4.5%) had an MCE. The proportion of patients with missing laboratory results and vital signs in REP and FHS were 1.6% (171) and 0.8% (330), respectively (eFigure 1 and eFigure 2 in the Supplement).

**Table 1.** Study Population

Variable	No. (%)	
	REP (n = 10 674)	FHS (n = 41 295)
**Demographic characteristic**		
Age, mean (SD), y^a^	59.4 (10.8)	57.4 (7.9)
Women	6324 (59.3)	21 975 (53.2)
Race/ethnicity		
White	9804 (91.9)	37 653 (91.2)
Black	179 (1.7)	1586 (3.8)
Asian	369 (3.5)	902 (2.2)
American Indian	21 (0.2)	240 (0.6)
Hawaiian	11 (0.1)	29 (0.1)
Unknown	290 (2.7)	885 (2.1)
**DSH risk score, mean (SD)**		
Type 2 diabetes		
Sum	5.4 (13.6)	12.2 (17.6)
Mean	0.4 (0.9)	0.9 (1.3)
Last observed	0.2 (0.4)	0.2 (0.8)
Hypertension		
Sum	38.2 (22.7)	47.3 (28.7)
Mean	2.5 (1.5)	3.4 (2.0)
Last observed	1.5 (1.6)	1.1 (2.3)
Hyperlipidemia		
Sum	19.5 (12.5)	18.4 (13.5)
Mean	1.3 (0.8)	1.3 (1.0)
Last observed	0.7 (1.1)	0.6 (1.2)
Obesity		
Sum	15.1 (12.1)	17.1 (12.0)
Mean	1.0 (0.8)	1.2 (0.9)
Last observed	0.7 (1.1)	0.3 (0.8)
**Comorbidities**		
Type 2 diabetes		
Most frequent	1733 (16.2)	15 716 (38.1)
Rolling	2664 (25.0)	26 438 (64.0)
Hypertension		
Most frequent	10 019 (93.9)	29 621 (71.7)
Rolling	10 575 (99.1)	41 218 (99.8)
Hyperlipidemia		
Most frequent	7678 (71.9)	21 839 (52.9)
Rolling	8806 (82.5)	28 563 (69.2)
Obesity		
Most frequent	4078 (38.2)	17 111 (41.4)
Rolling	4986 (46.7)	26 198 (63.4)
**Medication classes**		
Frequency, mean (SD), No.	23.2 (22.3)	14.0 (0.2)
ACE inhibitor	3416 (32.0)	25 968 (62.9)
Calcium channel blocker	2192 (20.5)	19 214 (46.5)
β-blocker	3958 (37.1)	30 437 (73.7)
Diuretic	1124 (10.5)	28 046 (67.9)
Statin	5687 (53.3)	31 294 (75.8)
α-blocker	1183 (11.1)	90 (0.2)
Angiotensin receptor blocker	1515 (14.2)	11 832 (28.6)
Other	262 (2.5)	12 440 (30.1)
Fibrate	478 (4.5)	4095.00 (9.92)
Sulfonylurea	660 (6.2)	7476 (18.1)
Renin inhibitor	7 (0.1)	70 (0.3)
Insulin	545 (5.1)	14 559 (35.2)
Cholesterol absorption inhibitor	587 (5.5)	4213 (10.2)
Metformin	1196 (11.2)	13 033 (31.6)
Dipeptidyl peptidase-4	124 (1.2)	2150 (5.2)
Meglitinide	23 (0.2)	203 (0.5)
Vasodilator	63 (0.6)	16 619 (40.2)
GLP-1 agonist	48 (0.5)	2070 (5.0)
Amylin	4 (<0.1)	52 (0.1)
SGLT-2 inhibitor	1 (<0.1)	608 (1.5)
**Frequency of laboratory results and vital signs, mean (SD)**		
Glycated hemoglobin	2.9 (6.4)	7.3 (9.3)
BNP	0.2 (1.2)	0.0 (0.0)
Creatinine	18.9 (28.5)	29.4 (39.1)
Cardiac troponin 1	0.1 (0.5)	0.0 (0.0)
Cardiac troponin T	2.5 (6.1)	0.0 (0.0)
Glucose		
Fasting	10.1 (11.3)	28.1 (38.1)
Random	0.1 (0.4)	33.6 (103.2)
Glomerular filtration rate	11.0 (27.5)	39.6 (66.8)
HDL cholesterol	8.9 (6.8)	8.9 (6.9)
LDL cholesterol	8.7 (6.7)	8.8 (6.8)
Total cholesterol	9.0 (6.9)	8.9 (6.9)
NT-proBNP	0.3 (1.6)	0.0 (0.0)
Triglycerides	8.8 (6.9)	9.0 (7.5)
BMI	18.2 (15.5)	38.3 (31.0)
Diastolic blood pressure	106.9 (204.6)	46.7 (39.5)
Height	20.4 (18.1)	0.0 (0.0)
Pulse	196.0 (618.4)	41.7 (35.9)
Systolic blood pressure	106.9 (204.5)	46.7 (39.5)
Weight	40.2 (41.3)	0.0 (0.0)
Respiration	61.1 (222.6)	0.0 (0.0)
**Laboratory results and vital signs, mean (SD)**		
Creatinine, mg/dL	1.0 (0.3)	6.1 (103.2)
Fasting glucose, mg/dL	104.1 (20.2)	119.9 (32.6)
Total cholesterol, mg/dL	193.3 (30.1)	178.8 (81.8)
BMI	28.4 (6.3)	35.1 (63.9)
Diastolic blood pressure, mm Hg	73.9 (7.0)	74.0 (6.7)
Pulse, bpm	73.3 (8.4)	74.5 (8.9)
Systolic blood pressure, mm Hg	127.1 (11.7)	128.0 (10.3)
**Last observed labs and vitals, mean (SD)**		
Creatinine, mg/dL	1.0 (0.3)	8.3 (146.9)
Fasting glucose, mg/dL	103.8 (20.2)	119.7 (33.9)
Total cholesterol, mg/dL	192.1 (30.0)	177.9 (152.3)
BMI	28.3 (6.2)	36.1 (76.8)
Diastolic blood pressure, mm Hg	73.6 (7.1)	73.8 (7.1)
Pulse, bpm	73.2 (8.6)	74.8 (9.3)
Systolic blood pressure, mm Hg	126.4 (11.8)	127.9 (10.8)
**Outcome**		
ACM	945 (8.9)	1857 (4.5)
MCE	787 (7.4)	3178 (7.7)

Abbreviations: ACE, angiotensin-converting enzyme; ACM, all-cause mortality; BMI, body mass index (calculated as weight in kilograms divided by height in meters squared); BNP, brain natriuretic peptide; bpm, beats per minute; DSH, disease severity hierarchy; FHS, Fairview Health Services; GLP-1, glucagon-like peptide 1; HDL, high-density lipoprotein; LDL, low-density lipoprotein; MCE, major cardiovascular event; NT-proBNP, N-terminal pro–brain natriuretic peptide; REP, Rochester Epidemiology Project; SGLT-2, sodium-glocuse cotransporter-2.

SI conversion factors: To convert creatinine to micromoles per liter, multiply by 88.4; fasting glucose to millimoles per liter, multiply by 0.055; and total cholesterol to millimoles per liter, multiply by 0.0259.

^a^ The index date for patients in REP was defined as age on January 1, 2004, and age on January 1, 2010, for FSH.

### Predicting ACM and MCE

Table 2 presents performance on REP and FHS. DSH-RS substantially outperformed COM. Specifically, models using DSH-RS achieved outstanding AUCs in predicting ACM at 60, 65, 75, and 80 years on REP data of 0.96 (95% CI, 0.94-0.97), 0.96 (95% CI, 0.95-0.98), 0.97 (95% CI, 0.96-0.98), and 0.98 (95% CI, 0.98-0.99), respectively, while models using COM produced modest AUCs of 0.67 (95% CI, 0.55-0.80), 0.66 (95% CI, 0.56-0.79), 0.64 (95% CI, 0.57-0.71), and 0.63 (95% CI, 0.54-0.70), respectively. Adding laboratory results and vital signs to COM (ie, COM + LB/VS) improved the performance of COM, resulting in AUCs between 0.73 and 0.87; however, the performance was still inferior to DSH-RS. External validation on FHS data demonstrated good performance for DSH-RS (AUCs, 0.77-0.81), outperforming COM (AUCs, 0.72-0.75) but with approximately the same performance as COM + LB/VS (AUCs, 0.80-0.82). A similar performance trend was observed for accuracy, sensitivity, specificity, and PPV (eg, age 80 years on REP for DSH-RS: accuracy, 0.96; 95% CI, 0.95-0.97; sensitivity, 0.99; 95% CI, 0.97-1.00; specificity, 0.95; 95% CI, 0.95-0.96; PPV, 0.55; 95% CI, 0.49-0.62; for COM: accuracy, 0.59; 95% CI, 0.51-0.67; sensitivity, 0.60; 95% CI, 0.51-0.70; specificity, 0.59; 95% CI, 0.50-0.67; PPV, 0.08; 95% CI, 0.05-0.10; for COM + LB/VS: accuracy, 0.72; 95% CI, 0.60-0.80; sensitivity, 0.58; 95% CI, 0.47-0.70; specificity, 0.73; 95% CI, 0.59-0.82; PPV, 0.11; 95% CI, 0.07-0.15; age 80 years on FHS for DSH-RS: accuracy, 0.74; sensitivity, 0.65; specificity, 0.74; PPV, 0.11; for COM: accuracy, 0.38; sensitivity, 0.88; specificity, 0.36; PPV, 0.06; for COM + LB/VS: accuracy, 0.35; sensitivity, 0.96; specificity, 0.33; PPV, 0.06).

**Table 2.** All-Cause Mortality Internal and External Validation

Model	Age, y	ACC	AUC	Sensitivity	Specificity	PPV
**Internal validation with cross-validation on REP: predictors in 2004-2010, follow-up in 2010-2015, mean (95% CI)**						
DSH-RS	60	0.91 (0.90-0.93)	0.96 (0.94-0.97)	0.98 (0.91-1.00)	0.91 (0.90-0.93)	0.10 (0.07-0.13)
	65	0.92 (0.90-0.93)	0.96 (0.95-0.98)	0.99 (0.94-1.00)	0.91 (0.90-0.93)	0.15 (0.10-0.19)
	75	0.94 (0.93-0.95)	0.97 (0.96-0.98)	0.98 (0.93-1.00)	0.94 (0.93-0.95)	0.35 (0.25-0.39)
	80	0.96 (0.95-0.97)	0.98 (0.98-0.99)	0.99 (0.97-1.00)	0.95 (0.95-0.96)	0.55 (0.49-0.62)
COM	60	0.62 (0.22-0.94)	0.67 (0.55-0.80)	0.49 (0.11-0.89)	0.62 (0.21-0.94)	0.02 (0.01-0.05)
	65	0.61 (0.38-0.93)	0.66 (0.56-0.79)	0.55 (0.17-0.85)	0.61 (0.38-0.94)	0.03 (0.01-0.05)
	75	0.54 (0.41-0.65)	0.64 (0.57-0.71)	0.64 (0.46-0.79)	0.54 (0.40-0.65)	0.05 (0.03-0.06)
	80	0.59 (0.51-0.67)	0.63 (0.54-0.70)	0.60 (0.51-0.70)	0.59 (0.50-0.67)	0.08 (0.05-0.10)
COM + LB/VS	60	0.80 (0.62-0.96)	0.86 (0.79-0.92)	0.68 (0.42-0.83)	0.80 (0.62-0.97)	0.06 (0.01-0.15)
	65	0.84 (0.70-0.96)	0.87 (0.82-0.93)	0.70 (0.48-0.87)	0.85 (0.70-0.97)	0.10 (0.03-0.23)
	75	0.79 (0.69-0.86)	0.79 (0.74-0.83)	0.64 (0.58-0.73)	0.80 (0.69-0.87)	0.11 (0.05-0.17)
	80	0.72 (0.60-0.80)	0.73 (0.67-0.78)	0.58 (0.47-0.70)	0.73 (0.59-0.82)	0.11 (0.07-0.15)
**External validation on FHS: predictors in 2010-2015, follow-up in 2015-2017, mean**						
DSH-RS	60	0.76	0.81	0.72	0.76	0.05
	65	0.75	0.80	0.68	0.76	0.07
	75	0.76	0.77	0.63	0.76	0.11
	80	0.74	0.77	0.65	0.74	0.11
COM	60	0.43	0.75	0.87	0.43	0.03
	65	0.41	0.74	0.88	0.39	0.04
	75	0.36	0.73	0.90	0.33	0.06
	80	0.38	0.72	0.88	0.36	0.06
COM + LB/VS	60	0.42	0.82	0.94	0.41	0.03
	65	0.46	0.81	0.92	0.45	0.04
	75	0.41	0.81	0.95	0.38	0.07
	80	0.35	0.80	0.96	0.33	0.06

Abbreviations: ACC, accuracy; AUC, area under the receiving operator characteristic curve; COM, comorbidities with medications; COM + LB/VS, COM with laboratory results and vital signs; DSH-RS, disease severity hierachy with risk scores only; FHS, Fairview Health Services; PPV, positive predictive value; REP, Rochester Epidemiology Project.

The eTable in the Supplement shows the results for predicting MCE on REP and FHS, in which we observed a similar strong performance of DSH-RS (internal AUCs, 0.75-0.79; external AUCs, 0.65-0.67), outperforming COM (internal AUCs, 0.54-0.58; external AUCs, 0.59-0.61) and COM + LB/VS (internal AUCs, 0.58-0.75; external AUCs, 0.57-0.59) on both the internal and external validation data sets.

**Figure 3.**
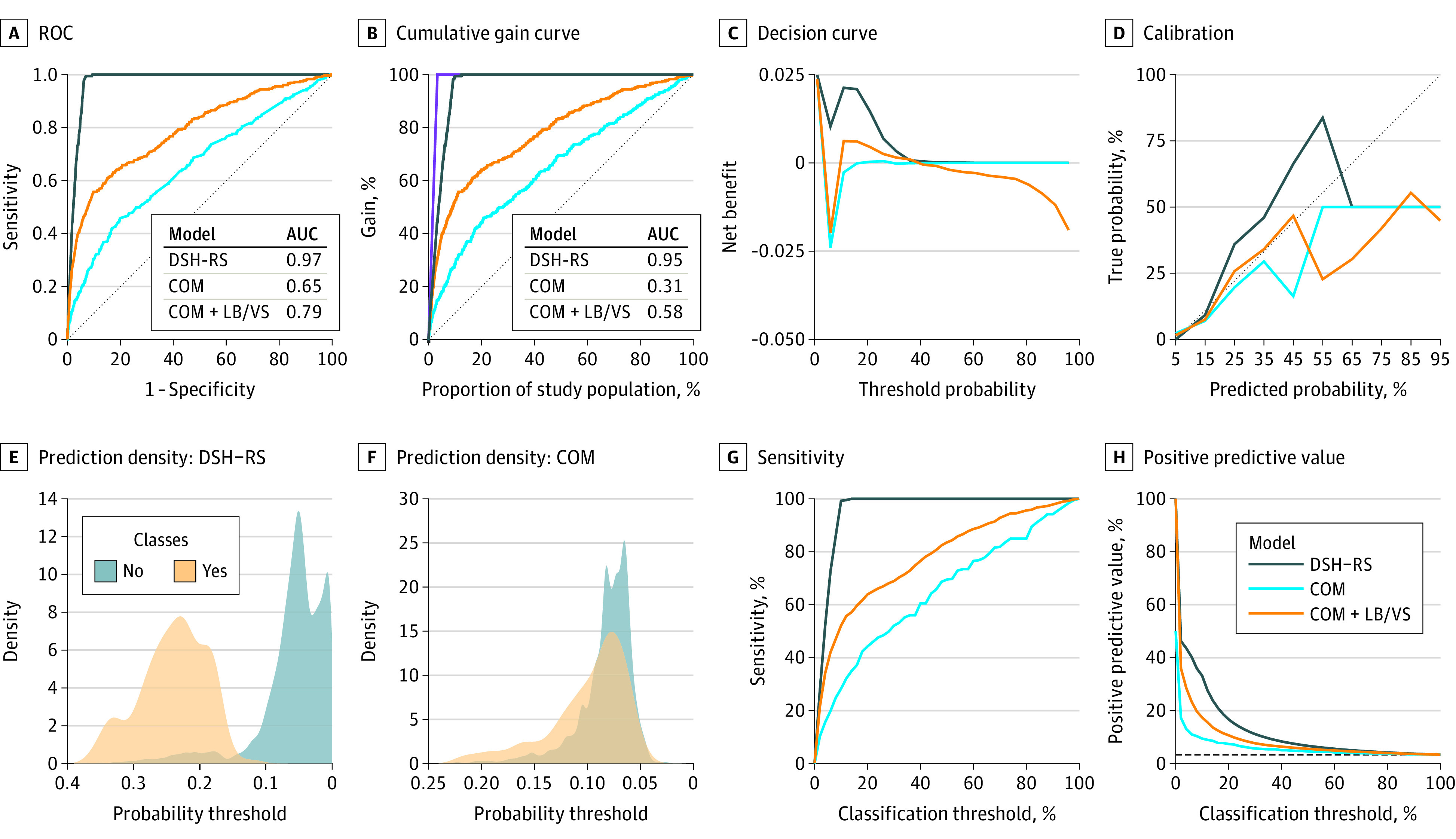
Internal Validation Performance Plots for Cox Proportional Hazard Models Predicting ACM at Age 75 Years B, The Gini score was computed by dividing the area between the gain curve and the random classifier (indicated by the dotted diagonal line) by the area between the perfect classifier (indicated by the purple curve) and the random classifier. D, the dotted diagonal line represents the line of perfect calibration. Systematic deviation (below or above) from the diagonal line indicates that the model might not reliably estimate event rates, leading to overestimation and underestimation. COM indicates model with only comorbidities and medication; COM + LB/VS, COM with laboratory results and vital signs; DSH-RS, disease severity hierarchy–risk score; ROC, receiver operating characteristic curve.

Figure 3 shows the receiver operating characteristic, cumulative gain, calibration, decision, prediction density, sensitivity, and PPV performance plots for predicting ACM at age 75 years for REP data. The AUC and Gini scores for DSH-RS (0.97 and 0.95, respectively) were considerably higher than those for COM (0.65 and 0.31, respectively) and COM + LB/VS (0.79 and 0.58, respectively) (Figure 3A and Figure 3B). The gain curve (Figure 3B) shows that if we were to select the top 10% of the entire population, representing 1067 patients, as high-risk for ACM at age 75 years based on DSH-RS, the sample will contain approximately 100% of high-risk patients. On the other hand, COM and COM + LB/VS will contain only 30.0% and 55.0% of high-risk cases, respectively. Similarly, the net benefit (Figure 3C), sensitivity (Figure 3G), and PPV (Figure 3H) curves of DSH-RS were higher than those for COM and COM + LB/VS. The class distributions in the prediction density plots (Figure 3E and Figure 3F), in which yes indicates death by age 75 years and no indicates survival older than 75 years, showed that DSH-RS accurately discriminated between low-risk and high-risk patients compared with COM. Complete graphic performance plots for ACE and MCE for REP data are available in eFigures 4 to 10 in the Supplement.

## Discussion

In this study, we introduced DSH, a novel representation of patient data that explores diseases and their known treatment pathways in a nested fashion. Unlike conventional methods of representing comorbidities as independent, time-invariant, and simple summaries, DSH offers explicit relationships for progression and severity of comorbidities as they relate to health status. We showed that these associations between comorbidities and their severity inform clinical risk stratification. This simple and expressive yet succinct representation of health status can be easily introduced to many clinical analysis processes and has the potential to greatly improve predictions. We demonstrated this capability by predicting age-time of ACM and MCE based on DSH representation of type 2 diabetes and its comorbidities. Models based on DSH representations outperformed models based on individual comorbidities, medications, laboratory results, and vital signs.

In the era of value-based health care, when practitioners are paid based on patient health outcomes, efficient risk stratification and patient management are becoming more important than ever. Health care organizations working to change their cost structure and improve outcomes must design interventions that target high-risk patients who need to be managed carefully and proactively. The first step to targeting high-risk patients is to identify them. Thus, implementing a platform to accurately stratify patients according to risk is the cornerstone of success for any population health management initiative.^27^ Several risk stratification systems, such as the Chronic Comorbidity Count, Adjusted Clinical Groups, Hierarchical Condition Categories, the Charlson-Elixhauser Comorbidity Index, and the Minnesota Health Care Home Tiering, among others, have been proposed.^28,29^ A commonality among these models is that they are based in some degree on comorbidity. This is not surprising given that understanding comorbid conditions and the heterogeneity exhibited by patients with MCCs is the first step in implementing effective population health management initiatives.^27,28,29^ Unfortunately, current representations of comorbidity indices do not reflect an individual’s comorbid disease history and severity or account for interactions with different treatment pathways.

This study was heavily driven by clinical domain knowledge and inspired by clinical thinking created by clinicians closely collaborating with data scientists. We aimed to make use of known relationships about specific diseases and their management and outcomes to improve the performance of machine learning models. By incorporating a priori domain knowledge into the data representations, we reduced the complexity of the problem, allowing the model to put observations in the clinical context, which otherwise would not be included in the modeling task. By explicitly modeling disease severity and treatment pathways, DSH-based models can improve human interpretability compared with competing methods.

Our ultimate goal was to generate information that can be used to develop EBPGs implementable in clinical decision support systems. EBPGs are series of interventions, each intervention being defined by a clinical action and a subpopulation for which this action would be most beneficial. Objective and accurate risk assessment tools are critically important for developing smart EBPGs and for counselling patients regarding the potential benefits and risks of different treatment strategies. The proposed DSH aimed to precisely identify patient subpopulations for which interventions may be useful.^26^

### Limitations

This study has limitations. First, the manual construction of DSH can be error prone and time consuming. How to automate the process, while remaining interpretable, is an open research question. Second, DSH uses retrospective EHR data, in which treatment pathways and disease intensity were determined based on whether a treatment was given. However, EHR data contain errors, and an erroneous treatment assignment may lead to inaccurate encoding of disease severity. Third, we have not investigated the generalizability of our methods to other complex comorbidities, nor have we investigated other functional relationships between the DSH nodes, other than doubling the score on branches to the right. It is not clear whether the results would remain consistent if other relationships (eg, an exponential relationship) were used. These are important open research questions to be investigated.

## Conclusions

In this study, we developed a novel data representation scheme and validated the added value in a risk prediction model. The model accurately predicted the age at which a patient is at risk of ACM or MCE substantially better than standard data presentations. DSH uses expert knowledge of known relationships within EHR data to capture disease severity in a natural and clinically meaningful way. The proposed DSH and corresponding risk scoring system can help to support critical decision-making, develop smart EBPGs, and enhance health care and disease management. It also has the potential to be used as a health care quality metric.

## Notes

Supplement. **eAppendix. ***International Classification of Diseases, Ninth Revision* (*ICD-9*) and *ICD-10-CM *codes **eTable. **Major Cardiovascular Event Internal and External Validation **eFigure 1. **Associations Between Missingness and Observations in Rochester Epidemiology Data **eFigure 2. **Associations Between Missingness and Observations in Fairview Health System Data **eFigure 3. **Disease Severity Hierarchy for Obesity **eFigure 4. **Predicted All-Cause Mortality at Age 60 Years **eFigure 5. **Predicted All-Cause Mortality at Age 65 Years **eFigure 6. **Predicted All-Cause Mortality at Age 80 Years **eFigure 7. **Predicted Major Cardiovascular Event at Age 60 Years **eFigure 8. **Predicted Major Cardiovascular Event at Age 65 Years **eFigure 9. **Predicted Major Cardiovascular Event at Age 75 Years **eFigure 10. **Predicted Major Cardiovascular Event at Age 80 Years
