# Supplementary material for: Development and Validation of a Risk Stratification Model Using Disease Severity Hierarchy for Mortality or Major Cardiovascular Event
Source: JAMA Netw Open. 2020 Jul 17;3(7):e208270. doi: 10.1001/jamanetworkopen.2020.8270 (PMC7368174; doi:10.1001/jamanetworkopen.2020.8270)
Supplement: Supplement. — eAppendix. International Classification of Diseases, Ninth Revision (ICD-9) and ICD-10-CM codes eTable. Major Cardiovascular Event Internal and External Validation eFigure 1. Associations Between Missingness and Observations in Rochester Epidemiology Data eFigure 2. Associations Between Missingness and Observations in Fairview Health System Data eFigure 3. Disease Severity Hierarchy for Obesity eFigure 4. Predicted All-Cause Mortality at Age 60 Years eFigure 5. Predicted All-Cause Mortality at Age 65 Years eFigure 6. Predicted All-Cause Mortality at Age 80 Years eFigure 7. Predicted Major Cardiovascular Event at Age 60 Years eFigure 8. Predicted Major Cardiovascular Event at Age 65 Years eFigure 9. Predicted Major Cardiovascular Event at Age 75 Years eFigure 10. Predicted Major Cardiovascular Event at Age 80 Years [file jamanetwopen-3-e208270-s001.pdf]

Supplementary Online Content

Ngufor C, Caraballo PJ, O'Byrne TJ, et al. Development and validation of a risk stratification model using disease severity hierarchy for mortality or major cardiovascular event. *JAMA Netw Open*. 2020;3(7):e208270. doi:10.1001/jamanetworkopen.2020.8270

eAppendix. *International Classification of Diseases, Ninth Revision* (*ICD-9*) and *ICD-10-CM* codes

eTable. Major Cardiovascular Event Internal and External Validation

eFigure 1. Associations Between Missingness and Observations in Rochester Epidemiology Data

eFigure 2. Associations Between Missingness and Observations in Fairview Health System Data

eFigure 3. Disease Severity Hierarchy for Obesity

eFigure 4. Predicted All-Cause Mortality at Age 60 Years

eFigure 5. Predicted All-Cause Mortality at Age 65 Years

eFigure 6. Predicted All-Cause Mortality at Age 80 Years

eFigure 7. Predicted Major Cardiovascular Event at Age 60 Years

eFigure 8. Predicted Major Cardiovascular Event at Age 65 Years

eFigure 9. Predicted Major Cardiovascular Event at Age 75 Years

eFigure 10. Predicted Major Cardiovascular Event at Age 80 Years

This supplementary material has been provided by the authors to give readers additional information about their work.

© 2020 Ngufor C et al. *JAMA Network Open.*

eAppendix. International Classification of Diseases, Ninth Revision (ICD-9) and ICD-10-CM codes

ICD 9/10-CM codes for the components of the major cardiovascular events outcome

1. Myocardial Infarction (ICD 9-CM): 410, 410.0, 410.00, 410.01, 410.02, 410.1, 410.10, 410.11, 410.12, 410.2, 410.20, 410.21, 410.22, 410.3, 410.30, 410.31, 410.32, 410.4, 410.40, 410.41, 410.42, 410.5, 410.50, 410.51, 410.52, 410.6, 410.60, 410.61, 410.62, 410.7, 410.70, 410.71, 410.72, 410.8, 410.80, 410.81, 410.82, 410.9, 410.90, 410.91, 410.92, 412
2. Stroke (ICD 9-CM): 430, 431, 432, 432.0, 432.1, 432.9, 997.02, 433, 433.0, 433.01, 433.1, 433.10, 433.11, 433.2, 433.21, 433.3, 433.31, 433.8, 433.81, 433.9, 433.91, 434, 434.0, 434.01, 434.1, 434.11, 434.9, 434.91, 436, 997.02
3. Percutaneous transluminal coronary angioplasty (PTCA):
    a. ICD 9-CM: V45.82
    b. ICD 10-CM: Z98.61, Z95.5
4. Use of cardiac device:
    a. cardiac pacemaker or an implantable cardioverter defibrillator
        i. ICD 9-CM: V45.01, V45.02, V53.31, V53.32, 996.01, 996.04
        ii. ICD 10-CM: Z45.010, Z45.018, Z45.02, Z95.0, Z95.810, T82.110A, T82.110D, T82.110S, T82.111A, T82.111D, T82.111S, T82.118A, T82.118D, T82.118S, T82.119A, T82.119D, T82.119S, T82.120A, T82.120D, T82.120S, T82.121A, T82.121D, T82.121S, T82.128A, T82.128D, T82.128S, T82.129A, T82.129D, T82.129S, T82.190A, T82.190D, T82.190S, T82.191A, T82.191D, T82.191S, T82.198A, T82.198D, T82.198S, T82.199A, T82.199D, T82.199S
    b. prosthetic heart valve
        i. ICD 9-CM: 996.02, 996.71, 996.09, 996.61, V42.2, V43.3, V53.3
        ii. ICD 10-CM: T82.01XA, T82.01XD, T82.01XS, T82.02XA,T82.02XD, T82.02XS,T82.03XA,T82.03XD, T82.03XS, T82.09XA, T82.09XD, T82.09XS, T82.221D, T82.221S, T82.222A, T82.222D, T82.222S, T82.223A, T82.223D, T82.223S, T82.228A, T82.228D, T82.228S, T82.6XXA, T82.6XXD, T82.6XXS,Z95.2, Z95.3, Z95.4
5. Coronary artery procedures
    a. ICD 9-CM: V45.81, V45.82, 414.02, 414.03, 414.04, 414.05, 414.06, 414.07, 996.03, 996.61
    b. ICD 10-CM: I25.700, I25.701, I25.708, I25.709, I25.710, I25.711, I25.718, I25.719, I25.720, I25.721, I25.728, I25.729, I25.730, I25.731, I25.738, I25.739, I25.750, I25.751, I25.758, I25.759, I25.760, I25.761, I25.768, I25.769, I25.790, I25.791, I25.798, I25.799, I25.810, I25.812, T82.211A, T82.211D, T82.211S, T82.212A, T82.212D, T82.212S, T82.213A, T82.213D, T82.213S, T82.218A, T82.218D, T82.218S, T82.7XXA, T82.7XXD, T82.7XXS, T82.817A, T82.817D, T82.817S, T82.818A, T82.818D, T82.818S, T82.827A, T82.827D, T82.827S, T82.828A, T82.828D, T82.828S, T82.837A, T82.837D, T82.837S, T82.838A, T82.838D, T82.838S, T82.847A, T82.847D, T82.847S, T82.848A, T82.848D, T82.848S, T82.855, T82.855A, T82.855D, T82.855S, T82.857A, T82.857D, T82.857S, T82.897A, T82.897D, T82.897S, T82.898A, T82.898D, T82.898S, T82.9XXA, T82.9XXD, T82.9XXS, Z95.1, Z95.5, Z95.9
6. Congestive heart failure
    a. ICD 9-CM: 428, 428.0, 428.1, 428.2, 428.20, 428.21, 428.22, 428.23, 428.3, 428.30, 428.31, 428.32, 428.33, 428.4, 428.40, 428.41, 428.42, 428.43, 428.9
    b. ICD 10-CM: I09.81, I11.0, I13.0, I13.2, I50.1, I50.20, I50.21, I50.22, I50.23, I50.30, I50.31, I50.32, I50.33, I50.40, I50.41, I50.42, I50.43, I50.8, I50.81, I50.810, I50.811, I50.812, I50.813, I50.814, I50.82, I50.83, I50.84, I50.89, I50.9
7. Ischemic heart disease
    a. ICD 9-CM: 411,411.0,411.1,411.8,411.81,411.89
    b. ICD 10-CM: I24.0,I24.1,I24.8,I24.9
8. Coronary artery disease
    a. ICD 9-CM: 414, 414.0, 414.00, 414.01, 414.02, 414.03, 414.04, 414.05, 414.06, 414.07, 414.1, 414.10, 414.11, 414.12, 414.19, 414.2, 414.3, 414.4, 414.8, 414.9

© 2020 Ngufor C et al. *JAMA Network Open.*

    b. ICD 10-CM: I25.10, I25.110, I25.111, I25.118, I25.119, I25.2, I25.3, I25.41, I25.42, I25.5, I25.6, I25.700, I25.701, I25.708, I25.709, I25.710, I25.711, I25.718, I25.719, I25.720, I25.721, I25.728, I25.729, I25.730, I25.731, I25.738, I25.739, I25.750, I25.751, I25.758, I25.759, I25.760, I25.761, I25.768, I25.769, I25.790, I25.791, I25.798, I25.799, I25.810, I25.811, I25.812, I25.82, I25.83, I25.84, I25.89, I25.9

9. Cardiomyopathy
    a. ICD 9-CM: 425, 425.0, 425.1, 425.11, 425.18, 425.2, 425.3, 425.4, 425.5, 425.7, 425.8, 425.9, 674.5, 674.50, 674.51, 674.52, 674.53, 674.54, 277.39
    b. ICD-10-CM: A36.81, B33.24, E85.4, I25.5, I42.0, I42.1, I42.2, I42.3, I42.4, I42.5, I42.6, I42.7, I42.8, I42.9, I43, O90.3
10. Cardiac arrest
    a. ICD 9-CM: 427.5, V12.53, 997.1, 779.85
    b. ICD 10-CM: I46.2, I46.8, I46.9, Z86.74, P29.81, I97.120, I97.121, I97.710, I97.711, O03.36, O03.86, O04.86, O07.36, O08.81, O29.111, O29.112, O29.113, O29.119
11. Angina
    a. ICD 9-CM: 411.1, 413, 413.0, 413.1, 413.9
    b. ICD 10-CM: I20.0, I20.1, I20.8, I20.9

© 2020 Ngufor C et al. *JAMA Network Open.*

eTable. Major Cardiovascular Event Internal and External Validation

Comparing the predictive ability of the different patient representations (Models) in discriminating MCE at age-time points 60, 65, 75, and 80 years. CPH models are trained for each representation through 10-fold cross-validation on the REP data and external validation on the FHS data. Data are expressed as mean (95% CI). The best AUC performance values are highlighted if bold font.

**Internal Validation with cross-validation on REP : Predictors in 2004 – 2010, follow up in 2010-2015**						
Model	Age	ACC	AUC	Sensitivity	Specificity	PPV
DSH-RS	60	0.64(0.60,0.69)	**0.79(0.75,0.83)**	0.78(0.61,0.87)	0.64(0.60,0.69)	0.06(0.04,0.08)
	65	0.72(0.60,0.84)	**0.79(0.76,0.83)**	0.70(0.53,0.84)	0.72(0.59,0.85)	0.10(0.06,0.16)
	75	0.72(0.66,0.81)	**0.77(0.73,0.80)**	0.67(0.48,0.77)	0.72(0.66,0.82)	0.13(0.10,0.16)
	80	0.69(0.62,0.75)	**0.75(0.72,0.79)**	0.67(0.52,0.79)	0.69(0.61,0.77)	0.13(0.11,0.15)
COM	60	0.69(0.63,0.73)	0.58(0.52,0.64)	0.43(0.34,0.51)	0.69(0.64,0.74)	0.04(0.03,0.06)
	65	0.67(0.60,0.78)	0.56(0.52,0.60)	0.43(0.27,0.50)	0.68(0.60,0.81)	0.05(0.03,0.07)
	75	0.59(0.51,0.73)	0.55(0.50,0.58)	0.47(0.27,0.62)	0.60(0.50,0.75)	0.07(0.05,0.08)
	80	0.56(0.49,0.63)	0.54(0.50,0.58)	0.50(0.37,0.59)	0.57(0.48,0.64)	0.08(0.06,0.09)
COM+LB/VS	60	0.70(0.62,0.82)	0.75(0.70,0.81)	0.68(0.57,0.85)	0.70(0.62,0.83)	0.07(0.05,0.10)
	65	0.71(0.63,0.77)	0.71(0.66,0.76)	0.60(0.46,0.76)	0.72(0.63,0.78)	0.08(0.05,0.11)
	75	0.61(0.53,0.71)	0.62(0.54,0.69)	0.56(0.36,0.72)	0.62(0.52,0.73)	0.08(0.05,0.10)
	80	0.61(0.54,0.68)	0.58(0.51,0.65)	0.51(0.32,0.68)	0.62(0.53,0.71)	0.09(0.06,0.10)
**External Validation on FSH : Predictors in 2010 – 2015, follow up in 2015-2017**						
DSH-RS	60	0.38	**0.67**	0.85	0.36	0.05
	65	0.41	**0.67**	0.82	0.39	0.07
	75	0.41	**0.65**	0.81	0.38	0.09
	80	0.40	**0.65**	0.82	0.37	0.09
COM	60	0.44	0.61	0.71	0.43	0.05
	65	0.44	0.60	0.70	0.42	0.06
	75	0.39	0.59	0.75	0.37	0.08
	80	0.37	0.59	0.76	0.34	0.08
COM+LB/VS	60	0.65	0.57	0.29	0.66	0.04
	65	0.65	0.58	0.28	0.67	0.05
	75	0.59	0.58	0.30	0.61	0.06
	80	0.59	0.59	0.30	0.61	0.06
MCE=major cardiovascular events, REP = Rochester Epidemiology Project, FHS = Fairview Health Services, DSH-RS = DSH risk scores only, COM = comorbidities + medications, COM + LB/VS = COM + labs and vital signs, ACC = accuracy, AUC = area under the ROC curve, CPH = Cox proportional hazard.						

© 2020 Ngufor C et al. *JAMA Network Open.*

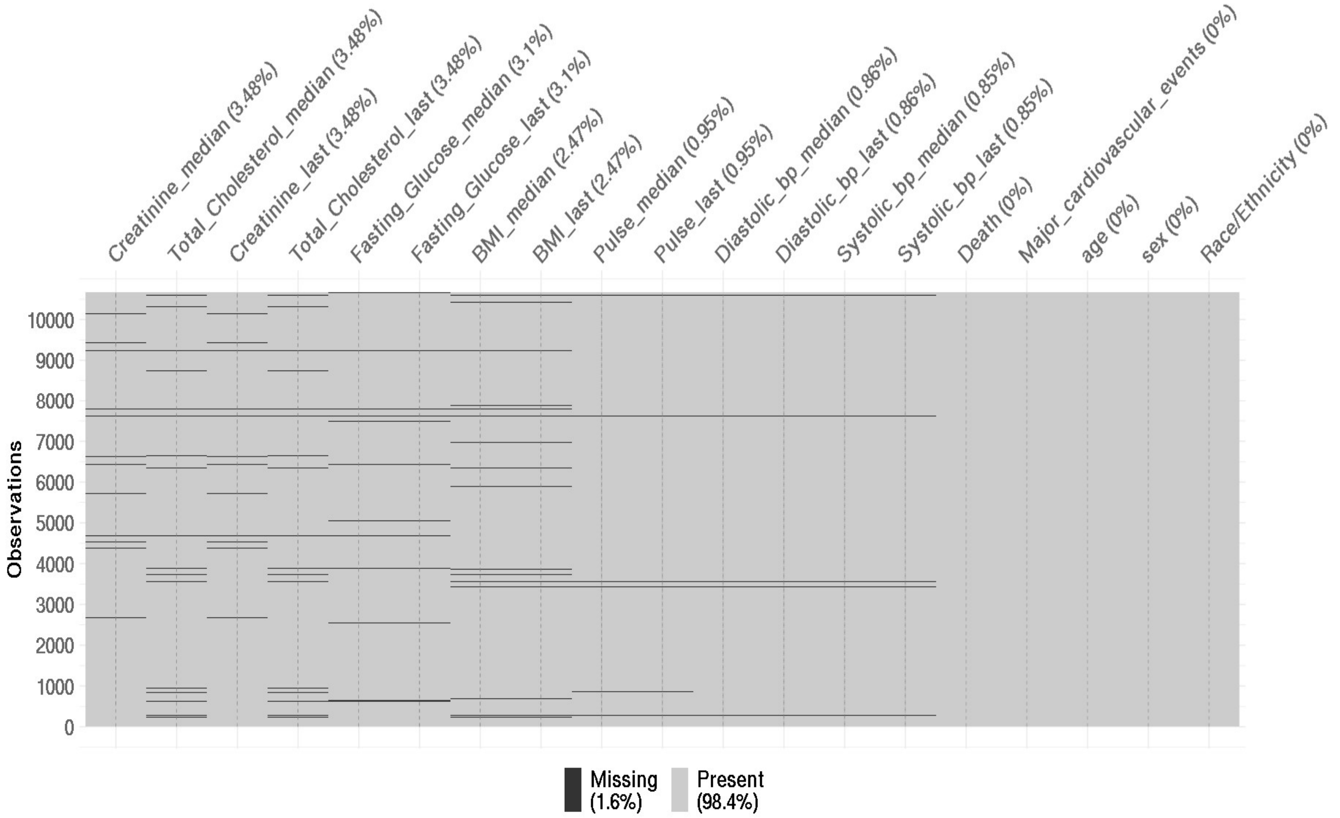

eFigure 1. Associations Between Missingness and Observations in Rochester Epidemiology Data

Patterns of missingness in the demographics, laboratory results (labs) and vital signs. The observations (row numbers in the data) are on the y-axis and the variables on the x-axis. Between 0.85% and 3.48% of observations in the Rochester Epidemiology Project (REP, n = 10,674) database are missing labs and vital signs. No clear relationship can be seen between the missing values.

© 2020 Ngufor C et al. *JAMA Network Open.*

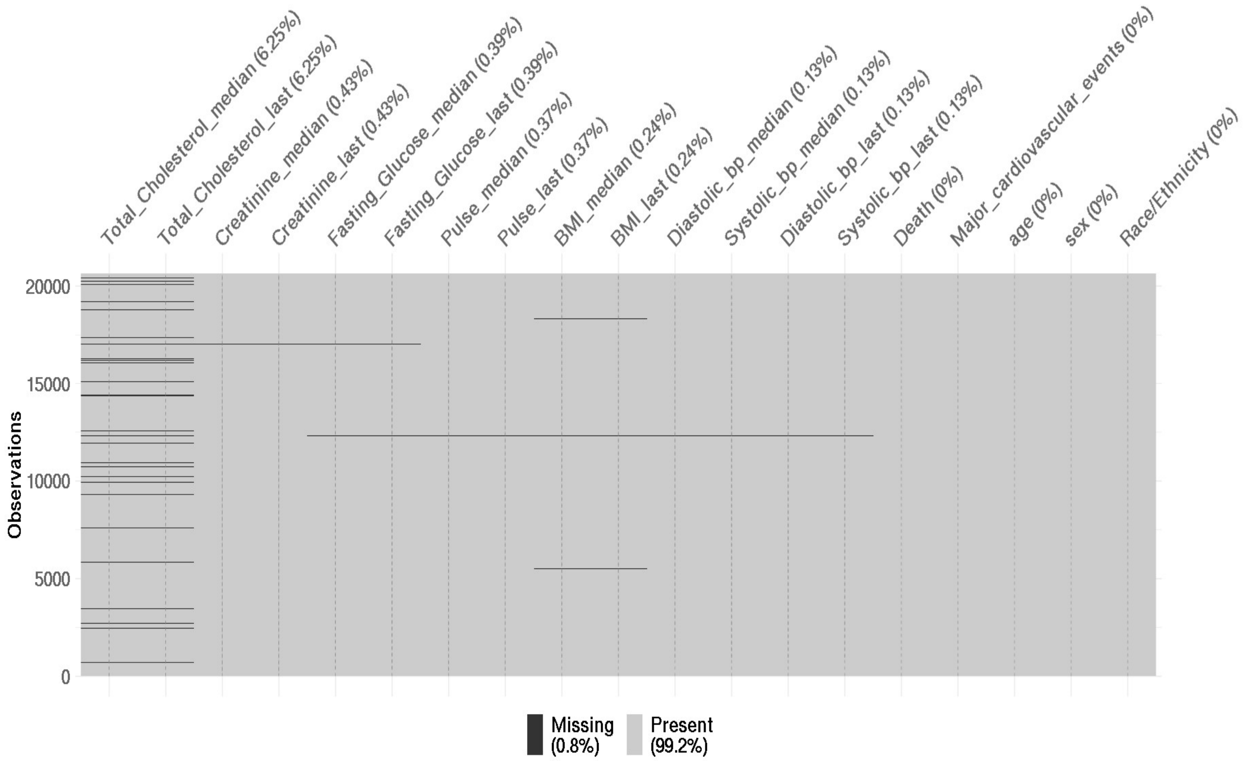

eFigure 2. Associations Between Missingness and Observations in Fairview Health System Data

Patterns of missingness in the outcomes, demographics, laboratory results (labs) and vital signs. The observations (row numbers in the data) are on the y-axis and the variables on the x-axis. Between 0.13% and 6.25% of observations in the Fairview Health Services (FHS, n=41,295) database are missing labs and vital signs. No clear relationship is observable between the missing values. Because of the large sample size of FHS, plotting all observations did not produce any visible pattern, so a random sample of 20,000 observations was plotted instead. However, the rates of missingness were the same as in the full sample.

© 2020 Ngufor C et al. *JAMA Network Open.*

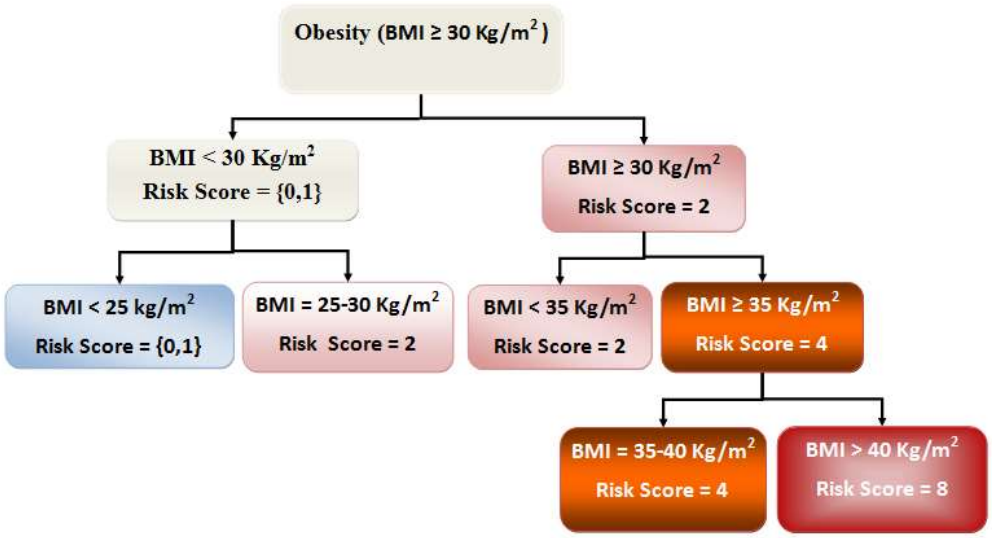

eFigure 3. Disease Severity Hierarchy for Obesity

An example of a 4-level DSH tree and corresponding risk score allocation for Obesity. At the root node, a patient is considered obese if the patients' BMI was ≥ 30 kg/m^2. Several different obesity control levels can be considered and risk scores allocated accordingly.

© 2020 Ngufor C et al. *JAMA Network Open.*

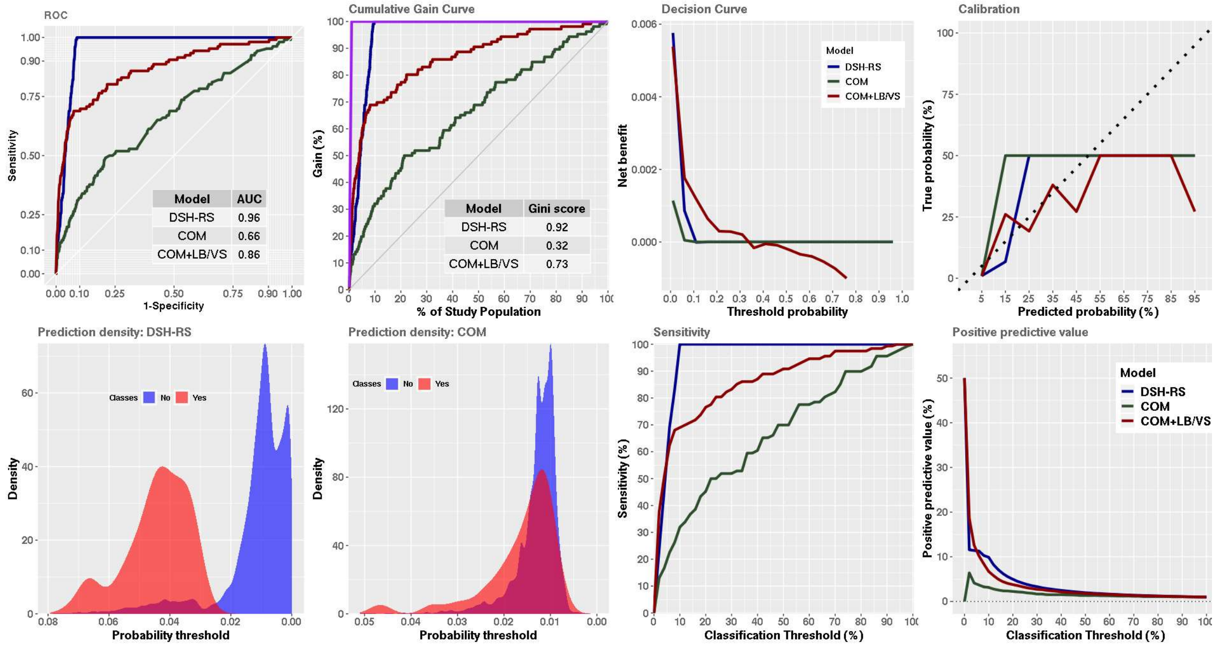

eFigure 4. Predicted All-Cause Mortality at Age 60 Years

Internal validation performance plots for the CPH models in predicting ACM at age 60 years. The blue, red and green curves represent the performance of DSH-RS, COM, and COM+LB/VS respectively. The AUC and Gini index of DSH-RS are significantly greater than that of COM and COM + LB/VS. Similarly, the ROC, gain, net benefit, sensitivity and positive predictive value curves for the DSH-RS model are significantly higher than those for COM and COM + LB/VS. The class distributions (Yes = dead at age 60, No = survive beyond 60 years) provided by the predicted density plot for DSH-RS are well separated, showing that DSH-RS accurately discriminates between low and high-risk patients compared to COM.

The Gini score was computed by dividing the area between the gain curve and the random classifier (gray diagonal line) by the area between the perfect classifier (purple curve) and the random classifier. The dotted diagonal line in the calibration plot represents the line of perfect calibration.

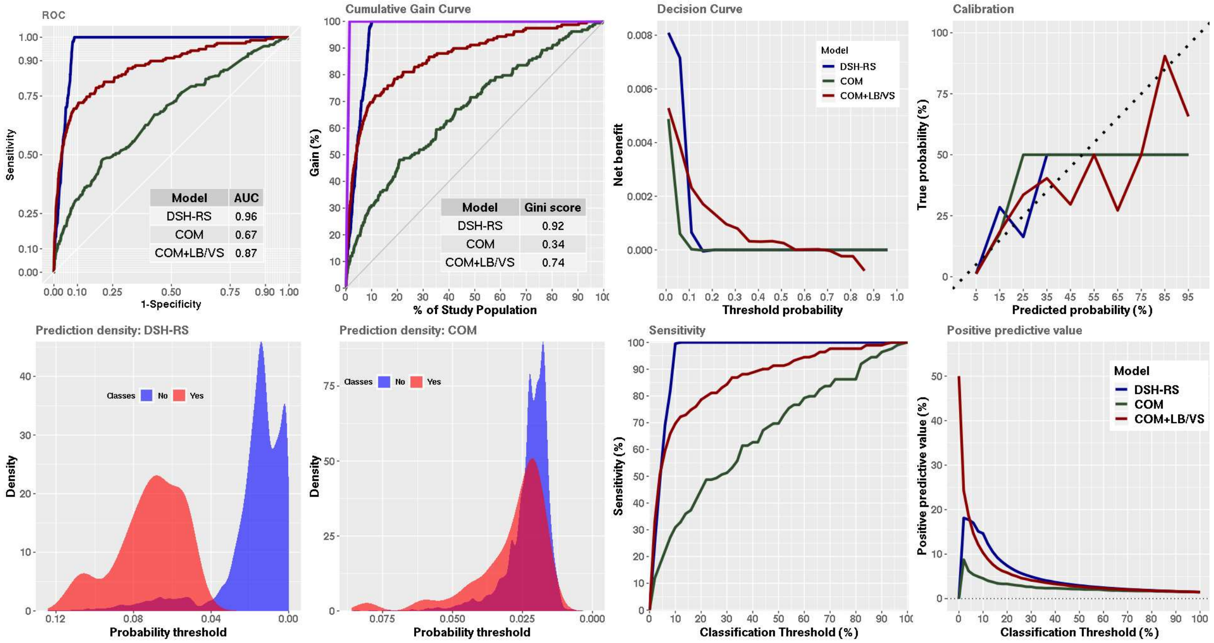

eFigure 5. Predicted All-Cause Mortality at Age 65 Years

Internal validation performance plots for the CPH models in predicting ACM at age 65 years. The blue, red and green curves represent the performance of DSH-RS, COM, and COM+LB/VS respectively. The AUC and Gini index of DSH-RS are significantly greater than that of COM and COM + LB/VS. Similarly, the ROC, gain, net benefit, sensitivity and positive predictive value curves for the DSH-RS model are significantly higher than those for COM and COM + LB/VS. The class distributions (Yes = dead at age 65, No = survive beyond 65 years) provided by the predicted density plot for DSH-RS are well separated, showing that DSH-RS accurately discriminates between low and high-risk patients compared to COM.

The Gini score was computed by dividing the area between the gain curve and the random classifier (gray diagonal line) by the area between the perfect classifier (purple curve) and the random classifier. The dotted diagonal line in the calibration plot represents the line of perfect calibration.

© 2020 Ngufor C et al. *JAMA Network Open.*

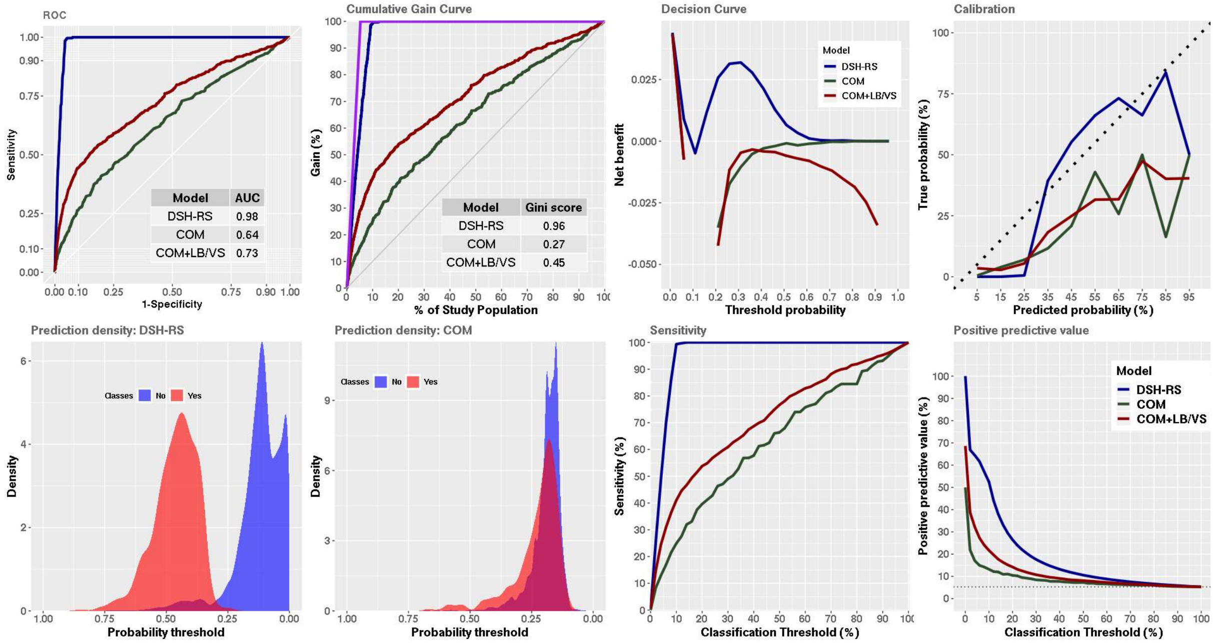

eFigure 6. **Predicted All-Cause Mortality at Age 80 Years**

Internal validation performance plots for the CPH models in predicting ACM at age 80 years. The blue, red and green curves represent the performance of DSH-RS, COM, and COM+LB/VS respectively. The AUC and Gini index of DSH-RS are significantly greater than that of COM and COM + LB/VS. Similarly, the ROC, gain, net benefit, sensitivity and positive predictive value curves for the DSH-RS model are significantly higher than those for COM and COM + LB/VS. The class distributions (Yes = dead at age 80, No = survive beyond 80 years) provided by the predicted density plot for DSH-RS are well separated, showing that DSH-RS accurately discriminates between low and high-risk patients compared to COM.

The Gini score was computed by dividing the area between the gain curve and the random classifier (gray diagonal line) by the area between the perfect classifier (purple curve) and the random classifier. The dotted diagonal line in the calibration plot represents the line of perfect calibration.

© 2020 Ngufor C et al. *JAMA Network Open.*

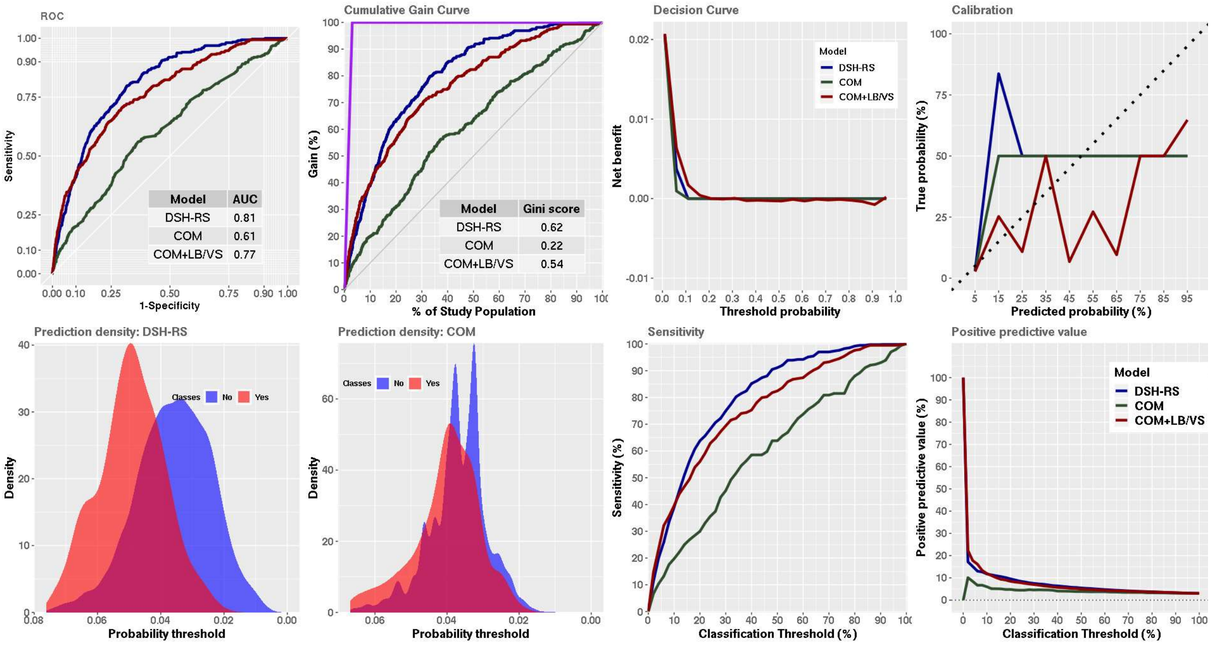

eFigure 7. Predicted Major Cardiovascular Event at Age 60 Years

Internal validation performance plots for the CPH models in predicting MCE at age 60 years. The blue, red and green curves represent the performance of DSH-RS, COM, and COM+LB/VS respectively. The AUC and Gini index of DSH-RS are significantly greater than that of COM and COM + LB/VS. Similarly, the ROC, gain, net benefit, sensitivity and positive predictive value curves for the DSH-RS model are all higher than those for COM and COM + LB/VS. The class distributions (Yes = MCE at age 60, No = no MCE beyond 60 years) for both DSH-RS and COM show some degree of overlap, however, the degree of overlap is very significant for COM. This illustrated that DSH-RS can more accurately discriminates between low and high-risk patients compared to COM.

The Gini score was computed by dividing the area between the gain curve and the random classifier (gray diagonal line) by the area between the perfect classifier (purple curve) and the random classifier. The dotted diagonal line in the calibration plot represents the line of perfect calibration.

© 2020 Ngufor C et al. *JAMA Network Open.*

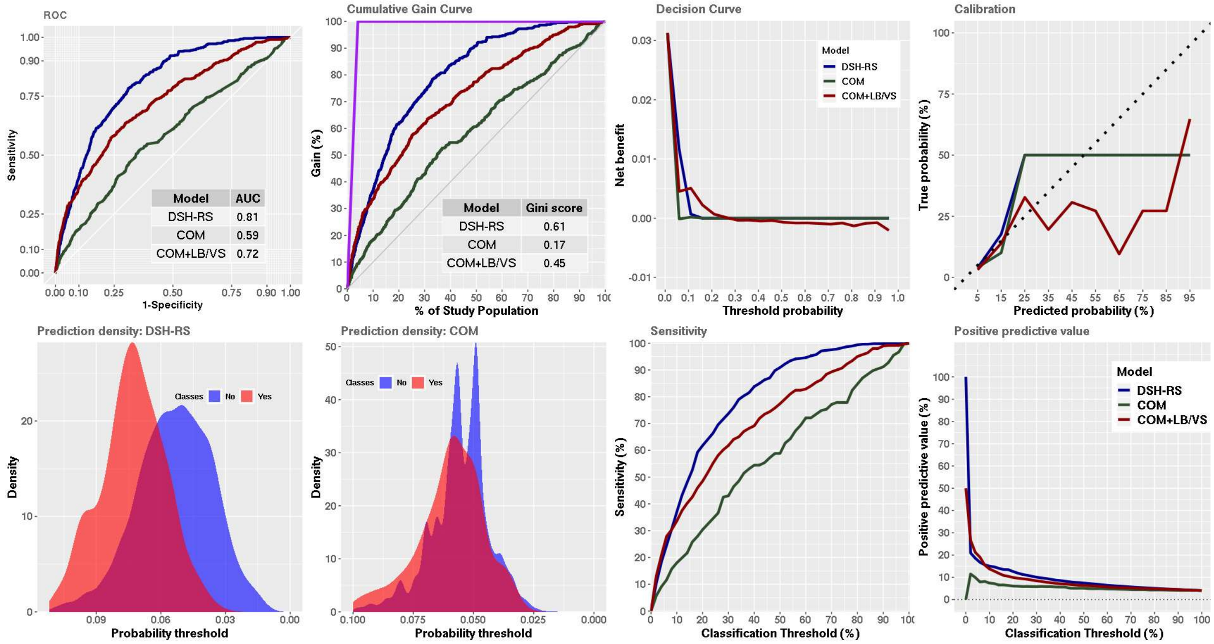

eFigure 8. Predicted Major Cardiovascular Event at Age 65 Years

Internal validation performance plots for the CPH models in predicting MCE at age 65 years. The blue, red and green curves represent the performance of DSH-RS, COM, and COM+LB/VS respectively. The AUC and Gini index of DSH-RS are significantly greater than that of COM and COM + LB/VS. Similarly, the ROC, gain, net benefit, sensitivity and positive predictive value curves for the DSH-RS model are all higher than those for COM and COM + LB/VS. The class distributions (Yes = MCE at age 65, No = no MCE beyond 65 years) for both DSH-RS and COM show some degree of overlap, however, the degree of overlap is very significant for COM. This illustrated that DSH-RS can more accurately discriminates between low and high-risk patients compared to COM.

The Gini score was computed by dividing the area between the gain curve and the random classifier (gray diagonal line) by the area between the perfect classifier (purple curve) and the random classifier. The dotted diagonal line in the calibration plot represents the line of perfect calibration.

© 2020 Ngufor C et al. *JAMA Network Open.*

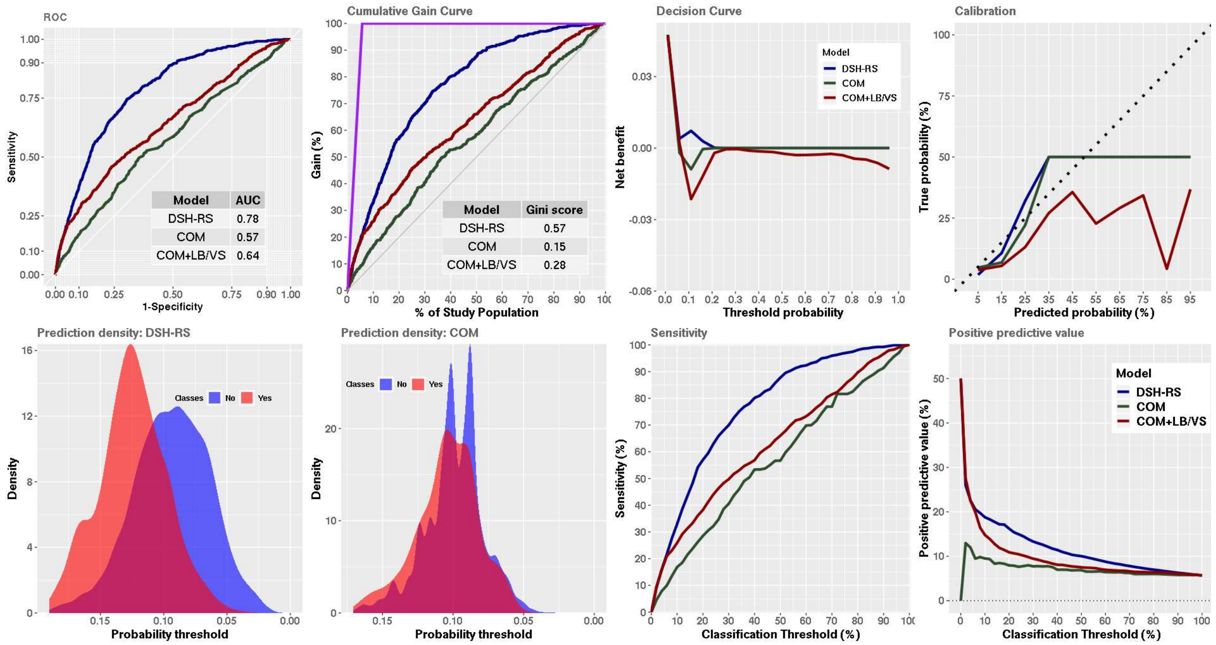

eFigure 9. Predicted Major Cardiovascular Event at Age 75 Years

Internal validation performance plots for the CPH models in predicting MCE at age 75 years. The blue, red and green curves represent the performance of DSH-RS, COM, and COM+LB/VS respectively. The AUC and Gini index of DSH-RS are significantly greater than that of COM and COM + LB/VS. Similarly, the ROC, gain, net benefit, sensitivity and positive predictive value curves for the DSH-RS model are all higher than those for COM and COM + LB/VS. The class distributions (Yes = MCE at age 75, No = no MCE beyond 75 years) for both DSH-RS and COM show some degree of overlap, however, the degree of overlap is very significant for COM. This illustrated that DSH-RS can more accurately discriminates between low and high-risk patients compared to COM.

The Gini score was computed by dividing the area between the gain curve and the random classifier (gray diagonal line) by the area between the perfect classifier (purple curve) and the random classifier. The dotted diagonal line in the calibration plot represents the line of perfect calibration.

© 2020 Ngufor C et al. *JAMA Network Open.*

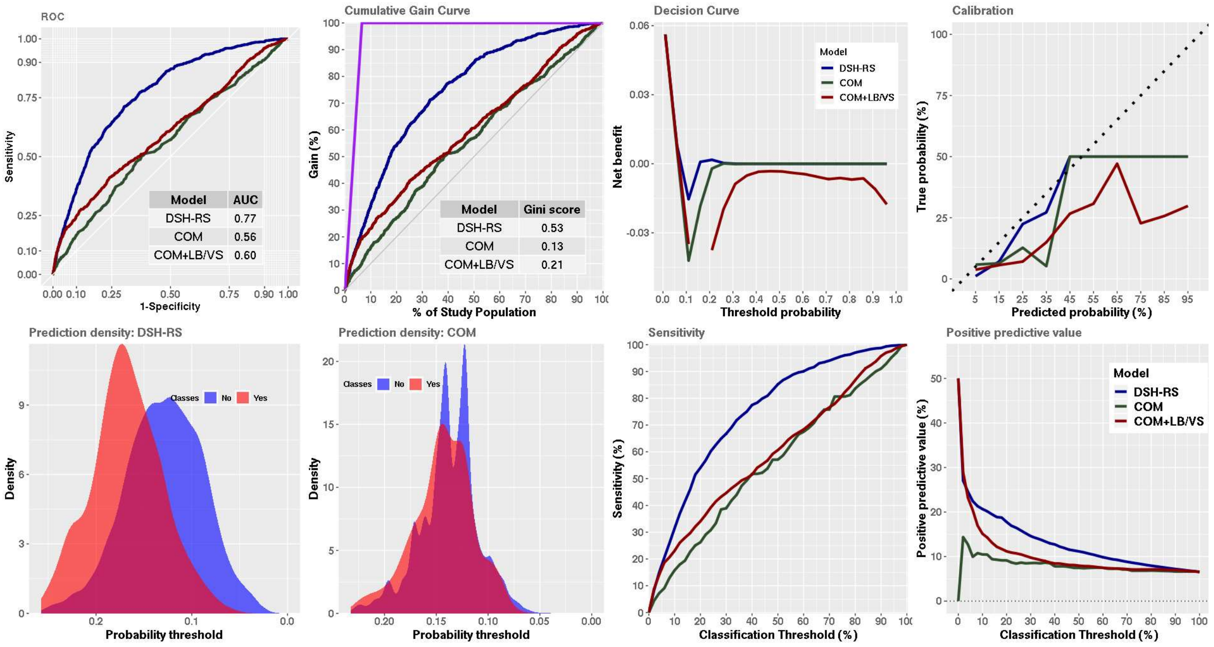

eFigure 10. Predicted Major Cardiovascular Event at Age at Age 80 Years

Internal validation performance plots for the CPH models in predicting MCE at age 80 years. The blue, red and green curves represent the performance of DSH-RS, COM, and COM+LB/VS respectively. The AUC and Gini index of DSH-RS are significantly greater than that of COM and COM + LB/VS. Similarly, the ROC, gain, net benefit, sensitivity and positive predictive value curves for the DSH-RS model are all higher than those for COM and COM + LB/VS. The class distributions (Yes = MCE at age 80, No = no MCE beyond 80 years) for both DSH-RS and COM show some degree of overlap, however, the degree of overlap is very significant for COM. This illustrated that DSH-RS can more accurately discriminates between low and high-risk patients compared to COM.

The Gini score was computed by dividing the area between the gain curve and the random classifier (gray diagonal line) by the area between the perfect classifier (purple curve) and the random classifier. The dotted diagonal line in the calibration plot represents the line of perfect calibration.

© 2020 Ngufor C et al. *JAMA Network Open.*
